# Comparison of Survival After Radical Hysterectomy or Radical Radiotherapy Among Patients with Different Histological Types of Locally Advanced Cervical Cancer: Analysis of SEER Data Using Propensity Score Matching

**DOI:** 10.3390/cancers18203371

**Published:** 2026-10-18

**Authors:** Yu Fan, Cheng-Wei Lian, Xiao-Yun Yang, Jin-Ke Li

**Affiliations:** 1 Department of Gynaecology and Obstetrics, West China Second Hospital, Sichuan University, Chengdu 610041, China; fanyu3@stu.scu.edu.cn (Y.F.);; 2 Key Laboratory of Birth Defects and Related Diseases of Women and Children, Sichuan University, Ministry of Education, Chengdu 610041, China

**Keywords:** locally advanced cervical cancer, radical hysterectomy, radiotherapy, survival, retrospective analysis

## Abstract

**Objective:** To compare survival of patients with different histological types of locally advanced cervical cancer after treatment with radical surgery or radical radiotherapy. **Methods:** Data for United States residents diagnosed with locally advanced cervical cancer between 2000 and 2022 were extracted from the Surveillance, Epidemiology, and End Results database. Overall survival and cancer-specific survival were compared using multivariable Cox proportional hazards regression. To minimize baseline differences, patients from each group were paired using propensity score matching. **Results:** Among the 1,363 patients with squamous cell carcinoma in the propensity score-matched cohort, surgery was associated with significantly better overall survival when disease was in stage IB3 (HR 0.70, 95% CI 0.50–0.97), but not when it was in other stages. Cancer-specific survival did not differ significantly between the two treatments at any disease stage. Among the 576 patients with adenocarcinoma in the propensity-score-matched cohort, surgery was associated with significantly better overall survival in stages IB3 (HR 0.46, 95% CI 0.28–0.77) or IIA2 (HR 0.38, 95% CI 0.15–0.96) or IIB involving tumors < 4 cm (HR 0.36, 95% CI 0.16–0.80). It was also associated with significantly better cancer-specific survival in stages IB3 (HR 0.51, 95% CI 0.29–0.90), IIA2 (HR 0.22, 95% CI 0.07–0.76) or IIB involving tumors < 4 cm (HR 0.21, 95% CI 0.07–0.62). **Conclusions:** Surgery may be associated with better overall survival than radiotherapy in stage IB3 squamous cell carcinoma. In the case of adenocarcinoma, surgery may be associated with better overall and cancer-specific survival than radiotherapy in stage IB3 or IIA2 and in stage IIB involving tumors < 4 cm.

## Simple Summary

Cervical cancer is the fourth most frequent cause of cancer-related deaths among women globally. Clinicians usually prefer to treat locally advanced disease using radical radiotherapy, with radical surgery considered an acceptable alternative. Whether this practice is valid across histological types and stages is unclear. Using data from the Surveillance, Epidemiology, and End Results database covering 2000–2022, we compared overall and cancer-specific survival between patients with locally advanced cervical cancer who were treated by surgery or radiotherapy. After propensity score matching, we found that surgery was associated with better overall survival in patients with squamous cell carcinoma in stage IB3 but not other stages. Surgery was associated with better overall and cancer-specific survival in patients with adenocarcinoma in stage IB3, IIA2 and in stage IIB involving tumors < 4 cm.

## 1. Introduction

Cervical cancer is the fourth most frequent cause of cancer-related deaths among women globally and the second most frequent cause among women in less developed countries [1]. Just over one third of women with cervical cancer are diagnosed after the disease has already advanced locally, and this rate is even higher among women in less developed countries [2]. While radical hysterectomy is an effective treatment for many women, it is associated with a risk of urological complications and is not an option for women who wish to bear children. Fortunately, radical radiotherapy can serve as a similarly effective and potentially safer alternative to surgery [3].

Both the National Comprehensive Cancer Network in the US and the International Federation of Gynaecology and Obstetrics (FIGO) recommend concurrent chemo- and radiotherapy for locally advanced cervical cancer in stages IB3, IIA2 or IIB, with surgery as an acceptable alternative in stages IB3 or IIA2 [4,5]. Whether these recommendations are equally valid for the two most frequent histological types of cervical cancer, squamous cell carcinoma and adenocarcinoma, is unclear. Studies have suggested that surgery may be superior to concurrent chemo- and radiotherapy for treating adenocarcinoma [6,7], and that the latter treatment may be less effective against adenocarcinoma than against squamous cell carcinoma [8,9,10,11].

The present study explored which primary treatment, radical surgery or radical radiotherapy, may lead to better survival for individuals with different histological types and stages of locally advanced cervical cancer.

## 2. Methods

This study was reported in accordance with the Strengthening the Reporting of Observational Studies in Epidemiology (STROBE) statement for observational cohort studies [12].

### 2.1. Study Population

Data were extracted from the “Surveillance, Epidemiology, and End Results” (SEER) database of US residents who were diagnosed between 2000 and 2022 with invasive cervical cancer originating in the cervix or endocervix of any of three histological types (squamous cell carcinoma, adenocarcinoma, or adenosquamous carcinoma) in stage T1b, T2a or T2b according to the American Joint Commission on Cancer system [13] and who underwent either radical radiotherapy ± concurrent chemotherapy or radical hysterectomy ± adjuvant therapy. This database is the largest population-based cancer registry in the US, covering just over one third of the population and 97% of all new cancer cases [14]. Since the SEER database contains public health data from which all personally identifiable elements have been removed, ethical review for the present work was considered unnecessary.

Individuals were excluded from this study if they were younger than 18 or older than 75 at diagnosis; if their cancer could not be assigned to stage IB3, IIA2 or IIB according to the 2018 FIGO scheme; if the cancer type was not confirmed histologically; if individuals showed nodal metastasis according to the American Joint Commission on Cancer system; or if data were insufficient to assign T, N or M stage. Individuals were also excluded if they had multiple primary malignant tumors.

### 2.2. Data Collection and Outcomes

Data were extracted from the release “Incidence–SEER Research Data, 17 Registries, Nov 2024 Sub (2000–2022)” using SEER*Stat 9.0.42.2 (https://seer.cancer.gov/data-software/, accessed on 1 October 2025). Individuals with adenocarcinoma or adenosquamous carcinoma were aggregated together because the two histological types are typically treated in similar ways, and patients show similar prognosis [4].

Clinicodemographic variables included age, year of diagnosis, and race/ethnicity (White, Black, or Other) as recorded in the database. Tumor characteristics included histological type, tumor grade, primary tumor size, and stage according to the 2018 FIGO scheme; if necessary, the original tumor staging was revised according to the TNM scheme based on pathology or imaging. The following treatments were recorded for each individual: conventional radical hysterectomy, modified radical hysterectomy, total hysterectomy, exenteration, other surgery, radical radiotherapy, adjuvant or concurrent chemotherapy, and adjuvant radiotherapy. Missing values were handled through complete-case deletion, with no imputation. Categories originally recorded in SEER as “Unknown” or “No/Unknown” were retained as a separate level in Cox regression models.

The outcomes of interest in this study were overall survival, defined as the time from diagnosis until any-cause death, and cervical-cancer-specific survival, defined as the time from diagnosis until death due to cervical cancer. Patients alive at the last follow up in 2022 were censored.

### 2.3. Statistical Analysis

Data were analyzed statistically using R 4.4.0 (https://cran.r-project.org/bin/windows/base/old/, accessed on 1 October 2025), and results associated with two-sided *p* < 0.05 were considered statistically significant. Depending on the analysis, age was treated as a continuous variable, in which case it was reported as median (interquartile range, IQR), or as a categorical variable, in which case it was dichotomized into ≤ 60 or > 60 years. Individuals were stratified according to whether they were diagnosed before or after 2014, when bevacizumab was approved by the US Food and Drug Administration.

Individuals were aggregated into one group who underwent any type of radical surgery, whether alone or followed by adjuvant chemo- or radiotherapy, and one group who underwent radical radiotherapy, whether alone or concurrent with chemotherapy. Within each group, individuals were stratified by revised 2018 FIGO stage (see Section 2.2). Intergroup differences in clinicodemographic variables were assessed for significance using the χ^2^ or Mann–Whitney U tests, as appropriate, while differences in Kaplan–Meier curves for overall and cancer-specific survival were assessed using the log-rank method.

Independent predictors of overall and cancer-specific survival for individuals in different disease stages were identified using multivariable Cox proportional hazards regression. Associations were quantified in terms of hazard ratios (HRs) and 95% confidence intervals (CIs) where appropriate. The differences in HRs across disease stages were unadjusted. We tested the proportional hazards assumption for each Cox regression using Schoenfeld residual tests and Kaplan–Meier curves, with *p* > 0.05 interpreted as lack of evidence of violation.

To minimize confounding due to patient differences at baseline, some analyses were performed after individuals in the surgery group had been matched to those in the radiotherapy group based on propensity scoring that took into account age at diagnosis, tumor size, tumor grade, and use of chemotherapy. The scoring was performed up to a 1:2 ratio using nearest-neighbor matching without replacement, with a caliper width of 0.1 times the standard deviation of the logit of the propensity score. However, the subsequent analyses did not account for the pairing structure.

We assessed the robustness of our results through various sensitivity analyses. First, we calculated between-group differences in restricted mean survival time at 60 months (ΔRMST_60_) and 120 months (ΔRMST_120_), together with 95% Cis, and these RMST differences were unadjusted. This survival metric is clinically interpretable and does not rely on the proportional hazards assumption [15]. Second, we calculated between-group survival differences separately for patients with adenocarcinoma or adenosquamous carcinoma instead of aggregating the two histological types together. Third, to address potential confounding by surgical staging, a sensitivity analysis was conducted excluding radiotherapy patients who underwent total hysterectomy, with all primary survival and propensity score matched models re-executed in this restricted cohort.

In additional sensitivity testing, we repeated analyses after stratifying individuals according to age at diagnosis, year of diagnosis, race/ethnicity, revised 2018 FIGO stage, tumor size, tumor grade or use of chemotherapy. Tumor size was used to stratify only individuals with stage IIB disease, because stages IB3 and IIA2 stipulate tumor size ≥ 4 cm. In the subgroup analysis of adjuvant radiotherapy, HRs were calculated for surgery with or without adjuvant radiotherapy, relative to radical radiotherapy.

All statistical analyses were conducted using R version 4.4.0, utilizing the following packages: tableone for baseline characteristics, MatchIt for propensity score matching, survival and survminer for survival and Cox regression analyses, and survRM2 for RMST calculations.

## 3. Results

### 3.1. Characteristics of the Study Population

**Figure 1:**
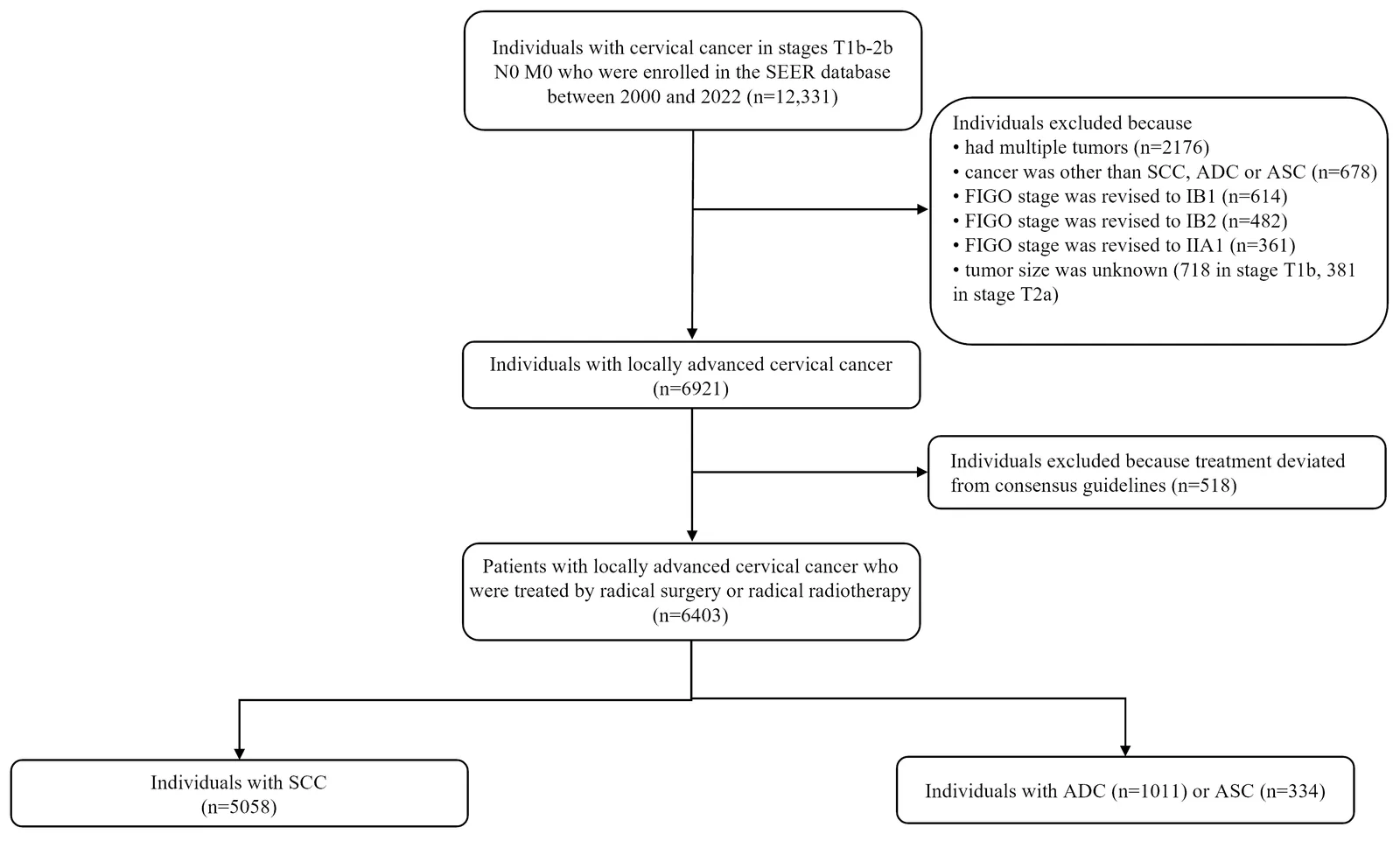
Flow diagram of patient selection. ADC, adenocarcinoma; ASC, adenosquamous carcinoma; FIGO, staging criteria from the International Federation of Gynecology and Obstetrics (2018 version); SCC, squamous cell carcinoma; SEER, “Surveillance, Epidemiology, and End Results” database.

Of the 12,331 individuals who were diagnosed with cervical cancer in stages T1b-2b with N0 and M0 during the enrollment period, we included 5058 with squamous cell carcinoma, 1011 with adenocarcinoma and 334 with adenosquamous carcinoma (Figure 1); the last two groups were aggregated together as “adenocarcinoma” in all analyses except as described in Section 3.3. Radiotherapy was the treatment for the vast majority of individuals, whether their cancer was squamous cell carcinoma or adenocarcinoma (Table 1); the standardized mean differences in baseline characteristics are presented in Supplementary Table S1. Of the 5520 patients in the radiotherapy arm, 773 (14%) underwent total hysterectomy, while more than 98% of the 883 patients in the surgery arm underwent radical hysterectomy. While those who underwent surgery were more likely to have tumors ≥ 4 cm, those who received radiotherapy were more likely to have stage IIB disease and to receive adjuvant chemotherapy.

### 3.2. Comparison of Survival After Surgery or Radiotherapy Among Individuals with Squamous Cell Carcinoma

**Figure 2:**
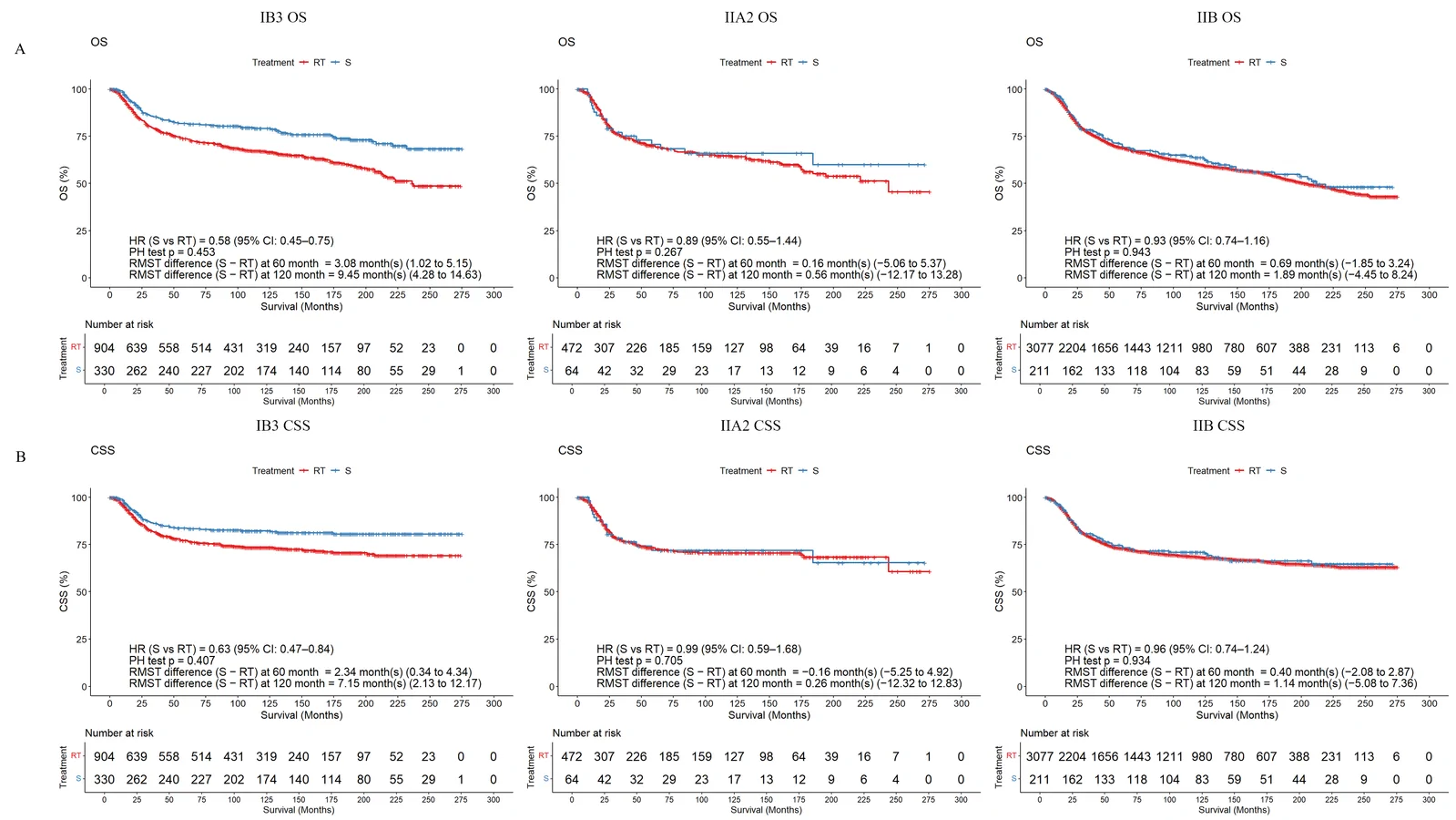

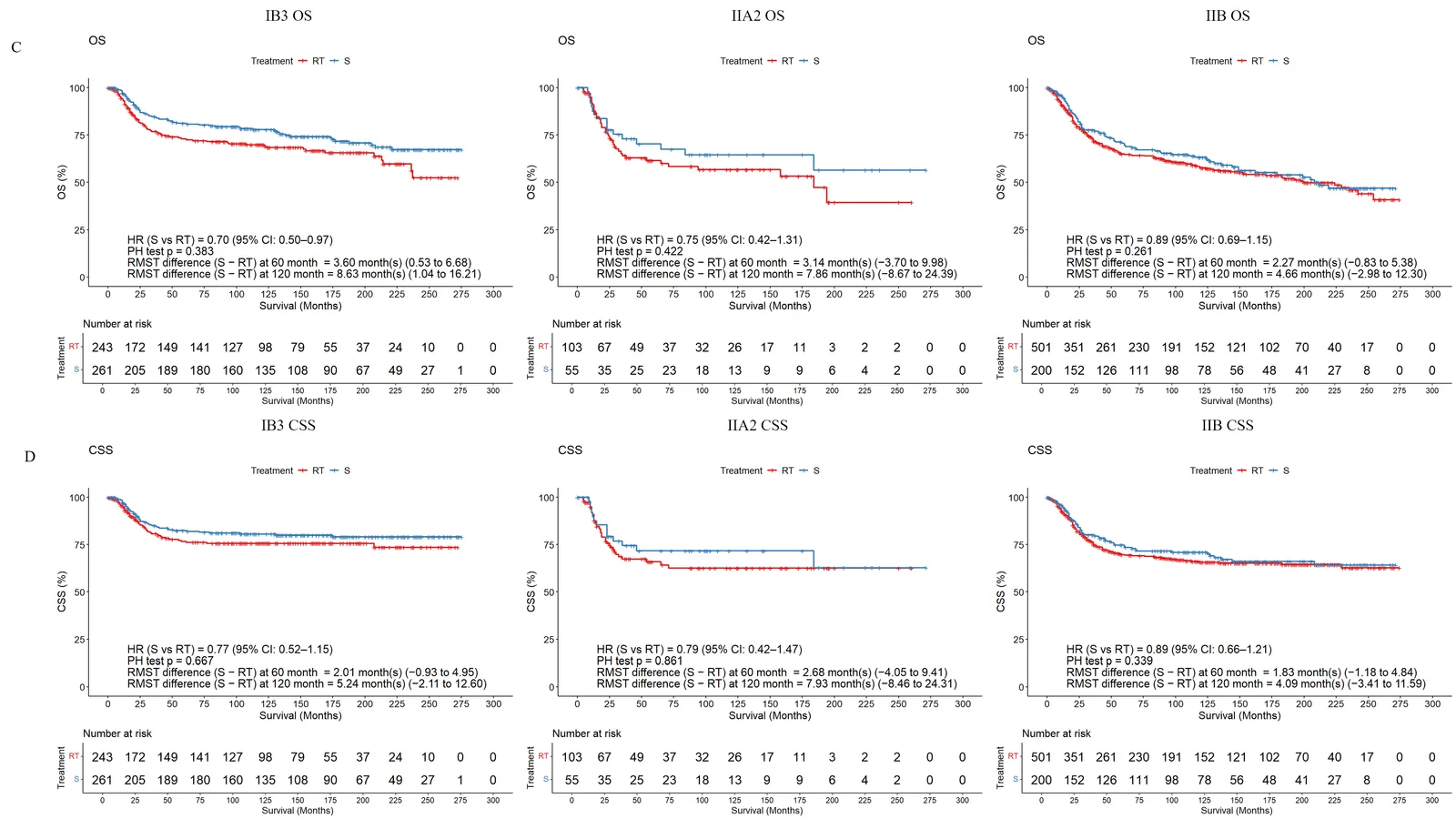
Analysis of overall survival (OS) and cervical cancer-specific survival (CSS) in individuals with locally advanced squamous cell carcinoma in the indicated stages at diagnosis. Analyses took into account (**A**,**B**) all patients or (**C**,**D**) subsets of patients matched to each other based on propensity scoring. HR, hazard ratio; PH, proportional hazards; RT, radiotherapy; RMST, restricted mean survival time; S, surgery.

Analysis of all enrolled individuals linked surgery to significantly higher overall survival among those with stage IB3 disease (HR 0.58, 95% CI 0.45–0.75; ΔRMST_60_, 3.08 months, 95% CI 1.02–5.15; ΔRMST_120_, 9.45 months, 95% CI 4.28–14.63; Figure 2A), but not among those in stages IIA2 or IIB (Figure 2A). Similarly, it linked surgery to significantly higher cancer-specific survival among those in stage IB3 (HR 0.63, 95% CI 0.47–0.84; ΔRMST_60_, 2.34 months, 95% CI 0.34–4.34; ΔRMST_120_, 7.15 months, 95% CI 2.13–12.17; Figure 2B), but not among those in stages IIA2 or IIB (Figure 2B).

Analysis of subsets of individuals matched to each other through propensity scoring confirmed a link between surgery and higher overall survival among those in stage IB3 (HR 0.70, 95% CI 0.50–0.97; ΔRMST_60_, 3.60 months, 95% CI 0.53–6.68; ΔRMST_120_, 8.63 months, 95% CI 1.04–16.21; Figure 2C), and the lack of such a link among those in stages IIA2 or IIB (Figure 2C). The analysis did not, however, confirm the link between surgery and higher cancer-specific survival (HR 0.77, 95% CI 0.52–1.15; ΔRMST_60_, 2.01 months, 95% CI −0.93–4.95; ΔRMST_120_, 5.24 months, 95% CI −2.11–12.60; Figure 2D), though it did confirm the lack of such a link among those in stages IIA2 or IIB (Figure 2D).

In sensitivity analyses excluding patients who underwent total hysterectomy in the radiotherapy group, the findings were consistent with the primary analyses (Supplementary Table S2).

In the absence of propensity score matching, exploratory subgroup analysis indicated that surgery was associated with better overall and cancer-specific survival than radiotherapy among individuals with any stage of squamous cell carcinoma when they were younger than 60 years at diagnosis, had grade 3–4 tumors, were of White ethnicity or received adjuvant radiotherapy (Table 2). Similar results were observed after propensity score matching, except that whether tumors were grade 3–4 did not influence cancer-specific survival.

In the absence of propensity score matching, multivariate Cox regression identified the following independent predictors of overall survival: surgery, age at diagnosis, ethnicity, tumor size in the case of stage IIB disease, FIGO stage, tumor grade, and use of chemotherapy (Supplementary Table S3). Independent predictors of cancer-specific survival were surgery, tumor size in the case of stage IIB disease, FIGO stage, and tumor grade. Similar results were observed after propensity score matching, except that neither type of survival was influenced by tumor grade, and cancer-specific survival was influenced only by tumor size in the case of stage IIB disease, FIGO stage, and use of chemotherapy.

### 3.3. Comparison of Survival After Surgery or Radiotherapy Among Individuals with Adenocarcinoma

We did not detect a statistically significant interaction of histological cancer type, whether adenocarcinoma or adenosquamous carcinoma, with treatment type on overall or cancer-specific survival (Supplementary Table S4), either before or after propensity score matching. Therefore, we conducted all subsequent analyses by aggregating patients with adenocarcinoma or adenosquamous carcinoma together under “adenocarcinoma”.

**Figure 3:**
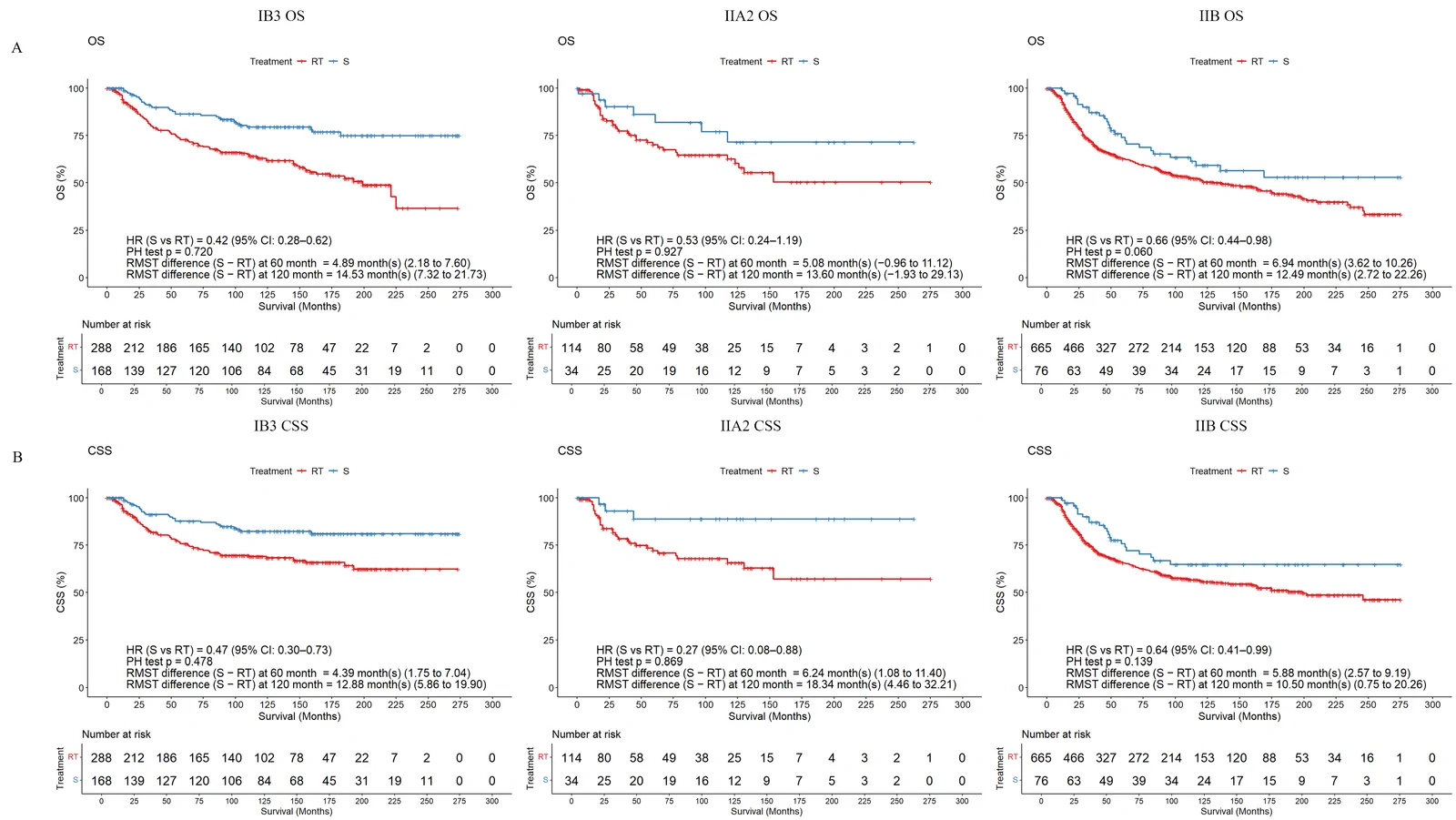

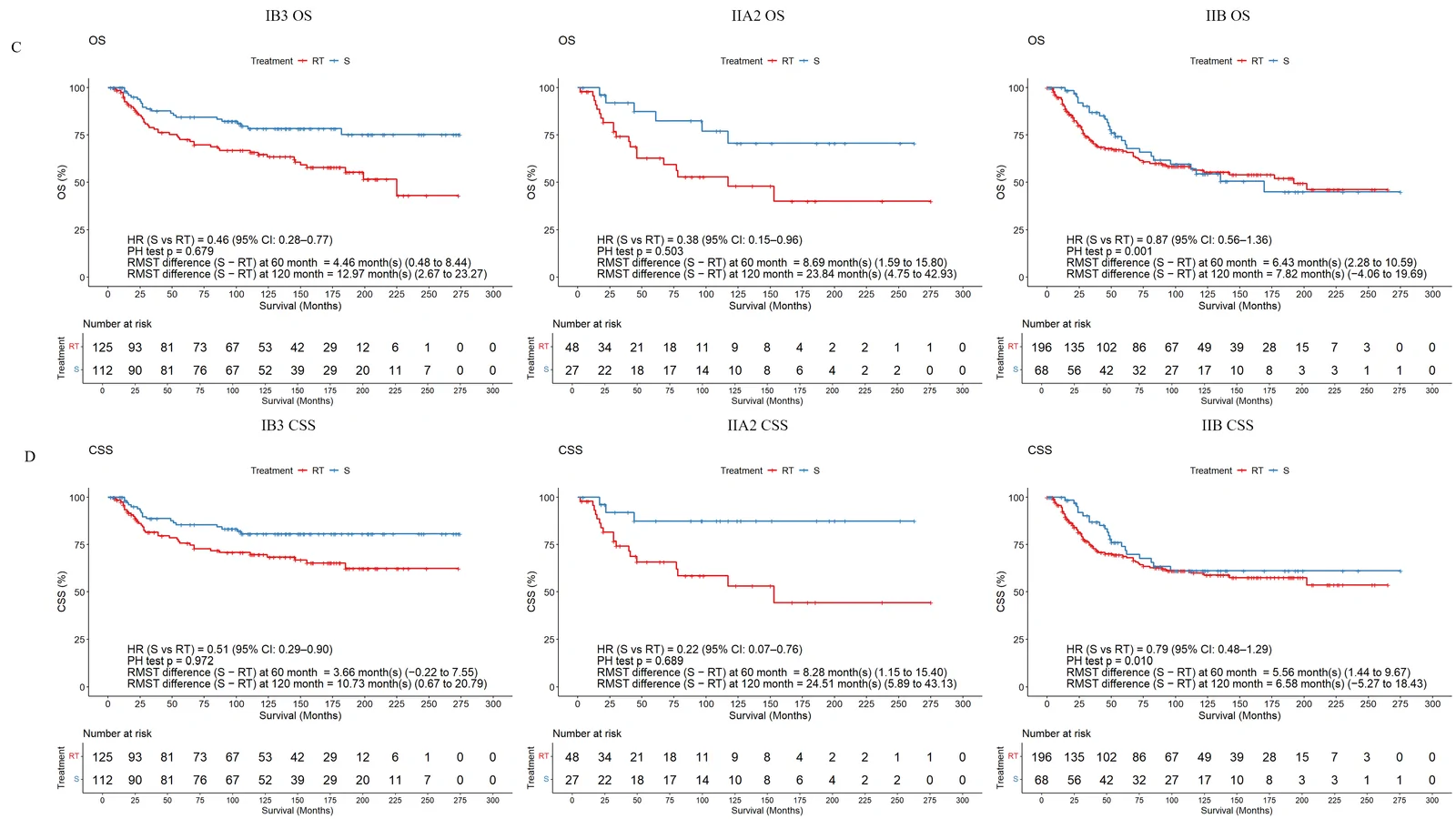
Analysis of overall survival (OS) and cervical cancer-specific survival (CSS) in individuals with locally advanced adenocarcinoma in the indicated stages at diagnosis. Analyses took into account (**A**,**B**) all patients or (**C**,**D**) subsets of patients matched to each other based on propensity scoring. HR, hazard ratio; PH, proportional hazards; RT, radiotherapy; RMST, restricted mean survival time; S, surgery.

Analysis of all enrolled individuals with adenocarcinoma linked surgery to significantly higher overall survival among those in stages IB3 (HR 0.42, 95% CI 0.28–0.62; ΔRMST_60_, 4.89 months, 95% CI 2.18–7.60; ΔRMST_120_, 14.53 months, 95% CI 7.32–21.73; Figure 3A) or IIB (HR 0.66, 95% CI 0.44–0.98; ΔRMST_60_, 6.94 months, 95% CI 3.62–10.26; ΔRMST_120_, 12.49 months, 95% CI 2.72–22.26; Figure 3A), but not those in stage IIA2 (HR 0.53, 95% CI 0.24–1.19; ΔRMST_60_, 5.08 months, 95% CI −0.96–11.12; ΔRMST_120_, 13.60 months, 95% CI −1.93–29.13; Figure 3A). In contrast, surgery was linked to significantly higher cancer-specific survival in all three stages, whether IB3 (HR 0.47, 95% CI 0.30–0.73; ΔRMST_60_, 4.39 months, 95% CI 1.75–7.04; ΔRMST_120_, 12.88 months, 95% CI 5.86–19.90; Figure 3B), IIA2 (HR 0.27, 95% CI 0.08–0.88; ΔRMST_60_, 6.24 months, 95% CI 1.08–11.40; ΔRMST_120_, 18.34 months, 95% CI 4.46–32.21; Figure 3B) or IIB (HR 0.64, 95% CI 0.41–0.99; ΔRMST_60_, 5.88 months, 95% CI 2.57–9.19; ΔRMST_120_, 10.50 months, 95% CI 0.75–20.26; Figure 3B).

Analysis of subsets of individuals matched to each other through propensity scoring confirmed a link between surgery and higher overall survival among those in stage IB3 (HR 0.46, 95% CI 0.28–0.77; ΔRMST_60_, 4.46 months, 95% CI 0.48–8.44; ΔRMST_120_, 12.97 months, 95% CI 2.67–23.27; Figure 3C). Instead, it suggested a link between surgery and higher overall survival among those in stage IIA2 (HR 0.38, 95% CI 0.15–0.96; ΔRMST_60_, 8.69 months, 95% CI 1.59–15.80; ΔRMST_120_, 23.84 months, 95% CI 4.75–42.93; Figure 3C). Similarly, the analysis indicated a link between surgery and higher cancer-specific survival among those in stages IB3 (HR 0.51, 95% CI 0.29–0.90; ΔRMST_60_, 3.66 months, 95% CI −0.22–7.55; ΔRMST_120_, 10.73 months, 95% CI 0.67–20.79; Figure 3D) and IIA2 (HR 0.22, 95% CI 0.07–0.76; ΔRMST_60_, 8.28 months, 95% CI 1.15–15.40; ΔRMST_120_, 24.51 months, 95% CI 5.89–43.13; Figure 3D). In the matched cohort, among patients with stage IIA2 disease (radiotherapy, n = 48; surgery, n = 27), overall survival events occurred in 20 versus 6 patients, and cancer-specific survival events occurred in 18 versus 3 patients, in the radiotherapy and surgery groups, respectively.

Among matched patients with stage IIB adenocarcinoma, the proportional hazards assumption was violated (overall survival, *p* = 0.001; cancer-specific survival, *p* = 0.010), indicating non-proportional hazards. The analysis revealed an association between surgery and early survival benefits, with a ΔRMST_60_ of 6.43 months (95% CI, 2.28–10.59) for overall survival and 5.56 months (95% CI, 1.44–9.67) for cancer-specific survival. However, this survival difference dissipated over longer follow-up, as indicated by non-significant HRs and ΔRMST_120_ for both overall survival (HR, 0.87; 95% CI, 0.56–1.36; ΔRMST_120_, 7.82 months; 95% CI, −4.06 to 19.69) and cancer-specific survival (HR, 0.79; 95% CI, 0.48–1.29; ΔRMST_120_, 6.58 months; 95% CI, −5.27 to 18.43).

In sensitivity analyses excluding patients who underwent total hysterectomy in the radiotherapy group, the findings were consistent with the primary analyses (Supplementary Table S5).

In the absence of propensity score matching, exploratory subgroup analysis indicated that surgery was associated with better overall and cancer-specific survival among those younger than 60 years, with grade 1–4 tumors, or with tumors smaller than 4 cm in the case of stage IIB disease (Table 3). Although use of chemotherapy was associated with overall survival benefits, tests for interaction between chemotherapy and treatment group were not statistically significant. A significant survival benefit was observed in those who underwent surgery compared to radical radiotherapy, regardless of whether they received adjuvant radiotherapy. The benefit of surgery for cancer-specific survival was observed among Whites and Blacks, whereas the benefit for overall survival appeared to be restricted to Whites. After propensity score matching, surgery was associated with better overall and cancer-specific survival among those who were younger than 60 years, were White, had grade 3–4 tumors, or received chemotherapy. Notably, for patients with adenocarcinoma, surgery conferred a consistent survival benefit irrespective of adjuvant radiotherapy status. Better overall and cancer-specific survival was observed in the case of stage IIB disease with tumors smaller than 4 cm. Among patients with stage IIB disease involving tumors < 4 cm (radiotherapy, n = 62; surgery, n = 40), overall survival events occurred in 25 versus 8 patients, and cancer-specific survival events occurred in 22 versus 4 patients, in the radiotherapy and surgery groups, respectively.

In the absence of propensity score matching, multivariate Cox modeling identified the following independent predictors of both overall and cancer-specific survival: surgery, age at diagnosis, ethnicity, tumor size in the case of stage IIB disease, FIGO stage, and tumor grade (Supplementary Table S6). After propensity score matching, the three variables of ethnicity, FIGO stage and tumor grade persisted as predictors of both types of survival, while age at diagnosis emerged as another predictor of overall survival.

In the matched cohort, among patients with stage IIA2 disease (radiotherapy, n = 48; surgery, n = 27), overall survival events occurred in 20 vs. 6 patients and cancer-specific survival events occurred in 18 vs. 3 patients in the radiotherapy and surgery groups, respectively. Among patients with stage IIB disease involving tumors < 4 cm (radiotherapy, n =62; surgery, n = 40), overall survival events occurred in 25 vs. 8 patients and cancer-specific survival events occurred in 22 vs. 4 patients in the radiotherapy and surgery groups, respectively.

## 4. Discussion

Our retrospective analysis of a substantial cohort of individuals treated for locally advanced cervical cancer in the US suggests that radical surgery may offer survival advantage over radical radiotherapy for at least several subpopulations. Among those with squamous cell carcinoma, surgery was associated with better overall survival when applied during stage IB3. Among those with adenocarcinoma, it was associated with better overall and cancer-specific survival when applied during stages IB3, IIA2, and stage IIB involving tumors < 4 cm. In contrast, surgery did not show a survival advantage over radiotherapy for individuals with squamous cell carcinoma in stage IIB, regardless of tumor size. The results in the propensity score-matched subset were broadly consistent with those observed in the primary cohort. However, certain associations were attenuated and lost statistical significance after matching, specifically cancer-specific survival for stage IB3 squamous cell carcinoma, as well as long-term overall and cancer-specific survival for stage IIB adenocarcinoma. Therefore, these results should be interpreted as exploratory and hypothesis-generating, warranting further validation in future studies.

Our study supports previous randomized controlled trials that reported comparable survival between individuals with squamous cell carcinoma in stage IIA2 or IIB who underwent either surgery or radiotherapy [16,17]. Our study extends that work by suggesting a survival advantage of surgery when the disease is in stage IB3. At the same time, the present work concurs with previous retrospective studies suggesting that surgery is associated with better survival than radiotherapy among individuals with adenocarcinoma in various stages [6,7,18]. We are unaware of randomized controlled trials that directly compared survival between individuals with adenocarcinoma who underwent surgery or radiotherapy.

Our results identify important differences in prognosis after radical surgery or radiotherapy between individuals with squamous cell carcinoma and adenocarcinoma. This may mean that patients should be managed differently depending on the histological type of cervical cancer. Indeed, adenocarcinoma is more likely to exhibit hematogenous dissemination [19,20] and resistance to radiotherapy [9] and chemotherapy [21]. These characteristics may help explain why overall survival is generally lower among patients with adenocarcinoma than among those with squamous cell carcinoma [8,9,10,22]. It may therefore be unwise to assume that the strong survival benefit of concurrent chemo- and radiotherapy against locally advanced cervical cancer in several randomized controlled trials [23,24,25] applies to patients with adenocarcinoma, given that 88–94% of patients in those trials had squamous cell carcinoma. A similar caveat applies to randomized trials reporting similar overall survival between patients undergoing surgery (after neoadjuvant chemotherapy) or radiotherapy, though progression-free survival was better in the radiotherapy arm [16,17].

A possible explanation for the prognostic differences between squamous cell carcinoma and adenocarcinoma in our SEER cohort is the greater radiosensitivity of squamous cell carcinoma [9,10,26]. We hypothesize that surgery may be sufficient for locoregional control of stage IB3 cancer of this histological type, when the disease is still confined to the cervix, whereas radiotherapy may be more effective after the tumor extends beyond the cervix to involve the vagina (stage IIA2) or parametrium (stage IIB). In our cohort, surgery and radiotherapy were associated with similar survival in stage IIB of squamous cell carcinoma, regardless of tumor size.

The greater radiosensitivity of squamous cell carcinoma may also help explain why individuals with adenocarcinoma, unlike those with squamous cell carcinoma, showed better survival after surgery than after radiotherapy when their disease was in stages IB3 and IIA2, as well as stage IIB involving tumors that were smaller than 4 cm. Although surgery was associated with an early survival benefit over radiotherapy in stage IIB adenocarcinoma, this benefit was no longer statistically significant for the overall stage IIB cohort at 120 months. This divergence can be explained by tumor size dynamics. Tumors < 4 cm are more amenable to complete resection with minimal residual disease, enabling a persistent long-term survival advantage [27]. For larger tumors, surgery rapidly reduces tumor burden; as adenocarcinoma is relatively radioresistant, the delayed response to radiotherapy further magnifies the early surgical benefit. Over the long term, however, larger tumors are strongly associated with distant micro-metastases [28] and incomplete resection. Consequently, despite superior short-term local control with surgery, the progression of distant metastases over time ultimately converges the long-term survival curves for the overall cohort.

Some of our exploratory subgroup analyses are also consistent with greater radiosensitivity of squamous cell carcinoma. Specifically, surgery was associated with better survival than radiotherapy among propensity score-matched patients with squamous cell carcinoma in the absence of chemotherapy or when coupled with adjuvant radiotherapy. For patients with squamous cell carcinoma, we hypothesize that radical radiotherapy, either alone or concurrent with chemotherapy, may be associated with similar prognosis as the combination of surgery followed by adjuvant chemotherapy, even when tumor burden is high [3,24,25]. We observed different outcomes for patients with adenocarcinoma: survival after surgery did not depend significantly on whether it was followed by adjuvant radiotherapy, and surgery was associated with better survival than radiotherapy only among patients who received chemotherapy. This suggests that the reported ability of chemotherapy to radiosensitize adenocarcinoma may be insufficient to make radiotherapy as effective as surgical resection [29].

We cannot exclude the possibility that factors other than radiosensitivity contributed to the survival advantage of surgery in our analyses. For example, adenocarcinoma often metastasizes to para-aortic lymph nodes [20,30], which means that staging of this histological type of cancer is more accurate when based on surgical biopsy, as in the case of patients treated surgically, than when based purely on imaging, as in the case of patients treated with radiotherapy. Therefore, our hypotheses about how the optimal treatment of cervical cancer depends on stage require verification in future work. Such work should, for example, verify our finding that when cancer was in stage IIB, tumor size emerged as an independent predictor of survival in the case of squamous cell carcinoma, but not adenocarcinoma, and this result was robust to propensity score matching. This difference implies that survival of adenocarcinoma patients in stage IIB is influenced more by the local extent of the tumor than by its size per se. Future studies should investigate whether surgery is associated with improved prognosis in patients with resectable stage IIB cervical adenocarcinoma for whom surgery is expected to achieve negative margins.

Regardless of whether the cervical cancer in our cohort was squamous cell carcinoma or adenocarcinoma, surgery was associated with better survival among those who were younger than 60 years at diagnosis, were of White ethnicity, or had grade 3–4 tumors. Such individuals are more likely to have relatively good health status at baseline and to enjoy higher socioeconomic status, which may make them more likely to complete both surgery and adjuvant therapy, thereby improving their survival [31,32,33].

Our findings should be interpreted with caution in light of several limitations. First, the retrospective design may limit the reliability of our results, for which we compensated through multivariable analysis and propensity score matching. Second, the SEER database lacked information on known prognostic factors such as lymphovascular invasion, tumor margin, response to radiotherapy, body mass index or surgical approach, so we could not assess their contribution to our results. For the same reason, we were unable to assess some clinically relevant outcomes beyond survival, such as treatment-related adverse events and health-related quality of life, which may differ between surgery and radiotherapy even when survival is comparable. Third, all patients in the surgery group were determined based on postoperative histopathological examination, which often leads to more advanced staging than imaging-based assessment [34]. This may have created a systematic staging bias in our analyses, so our findings need to be validated in the clinic, where treatment strategy is usually decided before pathological staging is available. Fourth, because the HRs and ΔRMSTs were unadjusted, residual confounding and potential selection bias cannot be entirely excluded, as certain covariates remained only partially balanced after propensity score matching. Furthermore, our matched analyses did not account for the matched-pair clustering structure. While this choice avoids certain modeling constraints, failing to account for pairing can potentially affect standard error estimation and variance structure. These methodological limitations should be considered when interpreting the comparative effectiveness of surgery versus radiotherapy. Fifth, in the radiotherapy group, 14% of patients underwent total hysterectomy, and this proportion increased after matching. The matched comparator may therefore be enriched for surgically staged or surgically treated patients and may not fully represent a definitive-radiotherapy population. We addressed this concern by performing a sensitivity analysis that excluded patients who underwent total hysterectomy in the radiotherapy group, and the results remained consistent with the primary analyses. Nevertheless, residual confounding by surgical staging, prior surgery, and unmeasured selection factors cannot be fully excluded, and this should be considered when interpreting the findings. Finally, although surgery showed a nominally significant survival advantage over radiotherapy in the IIA2 subgroup (Figure 3C,D) and stage IIB disease involving tumors < 4 cm (Table 3), the number of events in the analysis was quite small and the 95% CI was extremely wide. Further study is needed to verify whether surgery can be recommended as primary treatment for IIA2 and stage IIB disease involving tumors < 4 cm. Finally, although we performed numerous subgroup analyses across multiple covariates, two survival outcomes, and matched and unmatched cohorts, we did not adjust for multiple comparisons. This may increase the risk of type I error, meaning that some nominally significant survival benefits may not, in fact, be real. Our findings should therefore be interpreted as exploratory and validated in future studies.

## 5. Conclusions

Our study suggests that surgery may be associated with better survival than radiotherapy against squamous cell carcinoma in the early stage IB3, and that its survival advantage over radiotherapy against adenocarcinoma extends to stages IB3, IIA2 and even IIB with tumors < 4 cm. After propensity score matching, our results suggest that surgery may be associated with better survival than radiotherapy in individuals who are younger than 60 years at diagnosis, are of White ethnicity, or have grade 3–4 tumors, regardless of histological cancer type. Future work should verify and extend our findings in an effort to optimize the personalized treatment of cervical cancer based on histological type and stage.

## Figures and Tables

**Table 1:** Baseline characteristics of enrolled individuals with locally advanced cervical cancer.

Characteristic	Squamous Cell Carcinoma						Adenocarcinoma					
	All Patients			Propensity Score-Matched			All Patients			Propensity Score-Matched		
	Radiotherapy	Surgery	*p*	Radiotherapy	Surgery	*p*	Radiotherapy	Surgery	*p*	Radiotherapy	Surgery	*p*
n	4453	605		847	516		1067	278		369	207	
Age, yr	48.00 [40.00, 58.00]	44.00 [37.00, 55.00]	<0.001	46.00 [37.00, 56.00]	45.00 [37.00, 56.00]	0.414	48.00 [41.00, 57.00]	45.00 [39.00, 52.00]	<0.001	46.00 [41.00, 54.00]	46.00 [39.00, 54.00]	0.793
Age at diagnosis, yr			0.004			0.716			0.001			1.000
<60	3462 (77.7)	502 (83.0)		688 (81.2)	424 (82.2)		854 (80.0)	247 (88.8)		316 (85.6)	177 (85.5)	
≥60	991 (22.3)	103 (17.0)		159 (18.8)	92 (17.8)		213 (20.0)	31 (11.2)		53 (14.4)	30 (14.5)	
Year of diagnosis			<0.001			0.004			0.011			0.270
2000–2014	3085 (69.3)	479 (79.2)		607 (71.7)	407 (78.9)		695 (65.1)	204 (73.4)		246 (66.7)	148 (71.5)	
2015–2022	1368 (30.7)	126 (20.8)		240 (28.3)	109 (21.1)		372 (34.9)	74 (26.6)		123 (33.3)	59 (28.5)	
Ethnicity			0.008			0.345			0.018			0.007
White	3170 (71.2)	463 (76.5)		624 (73.7)	389 (75.4)		812 (76.1)	225 (80.9)		279 (75.6)	170 (82.1)	
Black	760 (17.1)	74 (12.2)		130 (15.3)	65 (12.6)		103 (9.7)	12 (4.3)		40 (10.8)	7 (3.4)	
Others	523 (11.7)	68 (11.2)		93 (11.0)	62 (12.0)		152 (14.2)	41 (14.7)		50 (13.6)	30 (14.5)	
Tumor size, cm			<0.001			0.713			<0.001			0.567
<4	599 (13.5)	86 (14.2)		132 (15.6)	81 (15.7)		163 (15.3)	44 (15.8)		62 (16.8)	40 (19.3)	
≥4	2850 (64.0)	478 (79.0)		642 (75.8)	397 (76.9)		700 (65.6)	225 (80.9)		295 (79.9)	158 (76.3)	
Unknown	1004 (22.5)	41 (6.8)		73 (8.6)	38 (7.4)		204 (19.1)	9 (3.2)		12 (3.3)	9 (4.3)	
FIGO stage			<0.001			<0.001			<0.001			<0.001
IB3	904 (20.3)	330 (54.5)		243 (28.7)	261 (50.6)		288 (27.0)	168 (60.4)		125 (33.9)	112 (54.1)	
IIA2	472 (10.6)	64 (10.6)		103 (12.2)	55 (10.7)		114 (10.7)	34 (12.2)		48 (13.0)	27 (13.0)	
IIB	3077 (69.1)	211 (34.9)		501 (59.1)	200 (38.8)		665 (62.3)	76 (27.3)		196 (53.1)	68 (32.9)	
Tumor Grade			<0.001			0.616			<0.001			0.731
1	144 (3.2)	5 (0.8)		5 (0.6)	5 (1.0)		139 (13.0)	53 (19.1)		61 (16.5)	36 (17.4)	
2	1537 (34.5)	243 (40.2)		349 (41.2)	221 (42.8)		302 (28.3)	120 (43.2)		141 (38.2)	77 (37.2)	
3–4	1582 (35.5)	325 (53.7)		448 (52.9)	258 (50.0)		307 (28.8)	91 (32.7)		133 (36.0)	80 (38.6)	
Unknown	1190 (26.7)	32 (5.3)		45 (5.3)	32 (6.2)		319 (29.9)	14 (5.0)		34 (9.2)	14 (6.8)	
Histology			NA			NA			0.016			0.016
Squamous cell carcinoma	4453 (100.0)	605 (100.0)		847 (100.0)	516 (100.0)		NA	NA		NA	NA	
Adenocarcinoma	NA	NA		NA	NA		818 (76.7)	193 (69.4)		280 (75.9)	137 (66.2)	
Adenosquamous carcinoma	NA	NA		NA	NA		249 (23.3)	85 (30.6)		89 (24.1)	70 (33.8)	
Surgery			<0.001			<0.001			<0.001			<0.001
Radical hysterectomy	0 (0.0)	595 (98.3)		0 (0.0)	506 (98.1)		0 (0.0)	274 (98.6)		0 (0.0)	203 (98.1)	
Total hysterectomy	530 (11.9)	0 (0.0)		170 (20.1)	0 (0.0)		243 (22.8)	0 (0.0)		107 (29.0)	0 (0.0)	
Exenteration	0 (0.0)	10 (1.7)		0 (0.0)	10 (1.9)		0 (0.0)	4 (1.4)		0 (0.0)	4 (1.9)	
Local tumor destruction/excision	473 (10.6)	0 (0.0)		95 (11.2)	0 (0.0)		109 (10.2)	0 (0.0)		46 (12.5)	0 (0.0)	
None	3450 (77.5)	0 (0.0)		582 (68.7)	0 (0.0)		715 (67.0)	0 (0.0)		216 (58.5)	0 (0.0)	
Chemotherapy			<0.001			<0.001			<0.001			0.022
Yes	4005 (89.9)	272 (45.0)		561 (66.2)	272 (52.7)		952 (89.2)	148 (53.2)		296 (80.2)	148 (71.5)	
No/Unknown	448 (10.1)	333 (55.0)		286 (33.8)	244 (47.3)		115 (10.8)	130 (46.8)		73 (19.8)	59 (28.5)	
Radiotherapy			<0.001			<0.001			<0.001			<0.001
Yes	4453 (100.0)	413 (68.3)		847 (100.0)	364 (70.5)		1067 (100.0)	192 (69.1)		369 (100.0)	164 (79.2)	
No/Unknown	0 (0.0)	192 (31.7)		0 (0.0)	152 (29.5)		0 (0.0)	86 (30.9)		0 (0.0)	43 (20.8)	

Values are n, n (%) or median [interquartile range], unless indicated otherwise. NA, not applicable.

**Table 2:** Analyses of overall and cervical-cancer-specific survival among individuals with squamous cell carcinoma stratified by different baseline characteristics, before and after propensity score matching.

Subgroup	Overall Survival				Cancer-Specific Survival			
	All Patients		Propensity Score-Matched		All Patients		Propensity Score-Matched	
	HR (95% CI)	p_interaction_	HR (95% CI)	p_interaction_	HR (95% CI)	p_interaction_	HR (95% CI)	p_interaction_
Age, yr								
<60	0.69 (0.58–0.82)	0.239	0.74 (0.60–0.93)	0.949	0.70 (0.57–0.85)	0.149	0.77 (0.60–0.99)	0.972
≥60	0.85 (0.61–1.17)		0.73 (0.50–1.09)		0.97 (0.65–1.45)		0.77 (0.47–1.26)	
Year of diagnosis								
2000–2014	0.64 (0.54–0.75)	0.014	0.69 (0.56–0.85)	0.220	0.66 (0.54–0.80)	0.010	0.70 (0.55–0.89)	0.118
2015–2022	1.09 (0.72–1.65)		0.96 (0.58–1.59)		1.25 (0.80–1.97)		1.11 (0.65–1.91)	
Ethnicity								
White	0.65 (0.54–0.78)	0.674	0.70 (0.56–0.88)	0.425	0.69 (0.56–0.85)	0.952	0.72 (0.55–0.93)	0.436
Black	0.79 (0.54–1.16)		0.75 (0.48–1.18)		0.89 (0.57–1.39)		0.81 (0.47–1.39)	
Others	0.92 (0.58–1.47)		1.03 (0.57–1.87)		0.91 (0.53–1.56)		1.15 (0.56–2.33)	
FIGO stage								
IB3	0.58 (0.45–0.75)	0.132	0.70 (0.50–0.97)	0.853	0.63 (0.47–0.84)	0.120	0.77 (0.52–1.15)	0.977
IIA2	0.89 (0.55–1.44)		0.75 (0.42–1.31)		0.99 (0.59–1.68)		0.79 (0.42–1.47)	
IIB	0.93 (0.74–1.16)		0.89 (0.69–1.15)		0.96 (0.74–1.24)		0.89 (0.66–1.21)	
Tumor grade								
1	0.82 (0.20–3.39)	0.880	NA	0.178	1.73 (0.41–7.35)	0.258	NA	
2	0.82 (0.64–1.04)		0.75 (0.56–1.02)		0.83 (0.63–1.11)		0.73 (0.52–1.04)	0.383
3–4	0.59 (0.48–0.74)		0.74 (0.57–0.96)		0.65 (0.51–0.83)		0.83 (0.61–1.11)	
Unknown	0.66 (0.35–1.23)		0.57 (0.25–1.26)		0.40 (0.15–1.08)		0.47 (0.15–1.50)	
Tumor size, cm *								
<4	1.13 (0.79–1.63)	0.300	1.19 (0.74–1.89)	0.208	1.21 (0.78–1.87)	0.413	1.07 (0.61–1.85)	0.552
≥4	0.89 (0.63–1.27)		0.84 (0.57–1.23)		0.97 (0.65–1.44)		0.90 (0.58–1.38)	
Unknown	1.01 (0.62–1.64)		0.77 (0.42–1.40)		0.95 (0.52–1.73)		0.94 (0.43–2.04)	
Chemotherapy								
Yes	0.77 (0.62–0.96)	0.006	0.82 (0.63–1.06)	0.088	0.81 (0.63–1.05)	0.086	0.88 (0.65–1.18)	0.099
No/Unknown	0.50 (0.39–0.64)		0.60 (0.45–0.79)		0.58 (0.43–0.77)		0.60 (0.43–0.84)	
Adjuvant Radiotherapy †								
Yes	0.69 (0.57–0.83)	0.987	0.70 (0.57–0.87)	0.591	0.71 (0.57–0.88)	0.523	0.69 (0.54–0.89)	0.223
No/Unknown	0.69 (0.53–0.89)		0.77 (0.57–1.03)		0.79 (0.59–1.06)		0.87 (0.63–1.21)	

CI, confidence interval; HR, hazard ratio; NA, not applicable. * This analysis was restricted to patients with stage IIB disease. † For the “Adjuvant Radiotherapy” subgroup, the HR compares patients who underwent surgery with or without adjuvant radiotherapy against the radical radiotherapy group, which did not receive adjuvant therapy.

**Table 3:** Analyses of overall and cervical-cancer-specific survival among individuals with adenocarcinoma stratified by different baseline characteristics, before and after propensity score matching.

Subgroup	Overall Survival				Cancer-Specific Survival			
	All Patients		Propensity Score-matched		All Patients		Propensity Score-matched	
	HR (95% CI)	p_interaction_	HR (95% CI)	p_interaction_	HR (95% CI)	p_interaction_	HR (95% CI)	p_interaction_
Age, yr								
<60	0.45 (0.33–0.60)	0.201	0.52 (0.36–0.75)	0.342	0.43 (0.31–0.59)	0.407	0.50 (0.34–0.74)	0.519
≥60	0.64 (0.38–1.10)		0.73 (0.39–1.39)		0.59 (0.30–1.17)		0.67 (0.30–1.46)	
Year of diagnosis								
2000–2014	0.43 (0.32–0.57)	0.384	0.56 (0.40–0.79)	0.998	0.43 (0.32–0.59)	0.873	0.54 (0.37–0.78)	0.828
2015–2022	0.58 (0.30–1.13)		0.58 (0.25–1.24)		0.40 (0.17–0.92)		0.47 (0.19–1.17)	
Ethnicity								
White	0.44 (0.32–0.59)	0.305	0.59 (0.41–0.85)	0.335	0.45 (0.33–0.63)	0.182	0.58 (0.39–0.86)	0.581
Black	0.41 (0.16–1.02)		0.39 (0.12–1.29)		0.21 (0.05–0.87)		0.36 (0.08–1.54)	
Others	0.71 (0.38–1.32)		0.78 (0.34–1.78)		0.61 (0.30–1.24)		0.56 (0.21–1.45)	
FIGO stage								
IB3	0.42 (0.28–0.62)	0.679	0.46 (0.28–0.77)	0.723	0.47 (0.30–0.73)	0.369	0.51 (0.29–0.90)	0.226
IIA2	0.53 (0.24–1.19)		0.38 (0.15–0.96)		0.27 (0.08–0.88)		0.22 (0.07–0.76)	
IIB	0.66 (0.44–0.98)		0.87 (0.56–1.36)		0.64 (0.41–0.99)		0.79 (0.48–1.29)	
Tumor grade								
1	0.34 (0.15–0.75)	0.467	0.56 (0.22–1.45)	0.857	0.38 (0.16–0.90)	0.697	0.65 (0.25–1.72)	0.892
2	0.48 (0.32–0.73)		0.62 (0.38–1.03)		0.46 (0.30–0.73)		0.60 (0.34–1.07)	0.383
3–4	0.53 (0.36–0.80)		0.59 (0.37–0.95)		0.50 (0.31–0.79)		0.53 (0.31–0.90)	
Unknown	0.27 (0.07–1.10)		0.22 (0.05–1.00)		0.17 (0.02–1.20)		0.14 (0.02–1.06)	
Tumor size, cm *								
<4	0.36 (0.17–0.75)	0.019	0.36 (0.16–0.80)	0.004	0.21 (0.08–0.59)	0.003	0.21 (0.07–0.62)	0.001
≥4	1.14 (0.66–1.94)		1.56 (0.85–2.84)		1.24 (0.71–2.16)		1.62 (0.87–3.05)	
Unknown	2.08 (0.76–5.68)		NA		2.31 (0.84–6.31)		NA	
Chemotherapy								
Yes	0.49 (0.35–0.69)	0.511	0.51 (0.35–0.74)	0.245	0.47 (0.32–0.69)	0.88	0.47 (0.31–0.70)	0.135
No/Unknown	0.42 (0.27–0.65)		0.79 (0.43–1.45)		0.49 (0.29–0.85)		0.87 (0.43–1.78)	
Adjuvant Radiotherapy †								
Yes	0.55 (0.41–0.73)	0.024	0.58 (0.41–0.82)	0.656	0.52 (0.38–0.73)	0.053	0.55 (0.38–0.81)	0.598
No/Unknown	0.28 (0.17–0.47)		0.50 (0.27–0.93)		0.27 (0.15–0.49)		0.45 (0.22–0.92)	

CI, confidence interval; HR, hazard ratio; NA, not applicable. * This analysis was restricted to patients with stage IIB disease. † For the “Adjuvant Radiotherapy” subgroup, the HR compares patients who underwent surgery with or without adjuvant radiotherapy against the radical radiotherapy group, which did not receive adjuvant therapy.

## Data Availability

The data in this study are publicly available from the National Cancer Institute’s Surveillance, Epidemiology, and End Results Program (https://seer.cancer.gov).

## Supplementary Materials

The following supporting information can be downloaded at: https://www.mdpi.com/article/10.3390/cancers18203371/s1, Supplementary Table S1. Standardized mean differences in baseline characteristics among enrolled patients with locally advanced cervical cancer before and after propensity score matching, before and after propensity score matching. Supplementary Table S2. Sensitivity analysis of treatment effect on survival in squamous cell carcinoma: including vs. excluding patients with total hysterectomy in the radiotherapy group. Supplementary Table S3. Multivariate regression to identify independent predictors of overall and cancer-specific survival among individuals with squamous cell carcinoma, before and after propensity score matching. Supplementary Table S4. Sensitivity analysis to assess interaction of histological type of cervical cancer (adenocarcinoma or adenosquamous carcinoma) with treatment type on survival. Supplementary Table S5. Sensitivity analysis of treatment effect on survival in adenocarcinoma: including vs. excluding patients with total hysterectomy in the radiotherapy group. Supplementary Table S6. Multivariate regression to identify independent predictors of overall and cancer-specific survival among individuals with adenocarcinoma, before and after propensity score matching.
