# Supplementary material for: Comparison of Survival After Radical Hysterectomy or Radical Radiotherapy Among Patients with Different Histological Types of Locally Advanced Cervical Cancer: Analysis of SEER Data Using Propensity Score Matching
Source: Cancers (Basel). 2026 Oct 18;18(20):3371. doi: 10.3390/cancers18203371 (PMC13702493; doi:10.3390/cancers18203371)
Supplement: Supplementary file 1 [file cancers-18-03371-s001.zip › cancers-4586087-supplementary/Supplementary Tables/Supplementary Table S1.pdf]

Supplementary Table S1. Standardized mean differences in baseline characteristics among enrolled patients with locally advanced cervical cancer before and after propensity score matching, before and after propensity score matching.

Characteristic	Squamous cell carcinoma						Adenocarcinoma					
	All patients			Propensity score-matched			All patients			Propensity score-matched		
	Radiotherapy	Surgery	SMD	Radiotherapy	Surgery	SMD	Radiotherapy	Surgery	SMD	Radiotherapy	Surgery	SMD
n	4,453	605		847	516		1,067	278		369	207	
Age, yr			0.236			0.047			0.278			0.012
Age at diagnosis, yr			0.132			0.024			0.245			0.004
Year of diagnosis			0.228			0.168			0.179			0.105
Ethnicity			0.142			0.082			0.211			0.293
Tumor size, cm			0.461			0.046			0.525			0.091
FIGO stage			0.789			0.468			0.782			0.445
Tumor Grade			0.665			0.074			0.706			0.100
Histology			0.000			0.000			0.164			0.215
Surgery			11.011			10.130			11.768			10.121
Chemotherapy			1.094			0.278			0.866			0.205
Radiotherapy			0.964			0.914			0.946			0.724

Values are n, n (%) or median [interquartile range], unless indicated otherwise. SMD, standardized mean differences.
