# Supplementary material for: Comparison of Survival After Radical Hysterectomy or Radical Radiotherapy Among Patients with Different Histological Types of Locally Advanced Cervical Cancer: Analysis of SEER Data Using Propensity Score Matching
Source: Cancers (Basel). 2026 Oct 18;18(20):3371. doi: 10.3390/cancers18203371 (PMC13702493; doi:10.3390/cancers18203371)
Supplement: Supplementary file 1 [file cancers-18-03371-s001.zip › cancers-4586087-supplementary/Supplementary Tables/Supplementary Table S2.pdf]

Supplementary Table S2. Sensitivity analysis of treatment effect on survival in squamous cell carcinoma: including vs. excluding patients with total hysterectomy in the radiotherapy group.

Outcome, subgroup, and hysterectomy in the radiotherapy group	FIGO stage	Number of		Hazard ratio	Difference in restricted mean survival time, months	
		Patients	Events		At 60 months	At 120 months
**Overall survival**						
All patients						
Included	IB3	1234	370	0.58 (0.45-0.75)	3.08 (1.02-5.15)	9.45 (4.28-14.63)
	IIA2	536	166	0.89 (0.55-1.44)	0.16 (-5.06-5.37)	0.56 (-12.17-13.28)
	IIB	3288	1248	0.93 (0.74-1.16)	0.69 (-1.85-3.24)	1.89 (-4.45-8.24)
Excluded	IB3	1001	319	0.49 (0.38-0.63)	4.81 (2.56-7.06)	13.92 (8.32-19.52)
	IIA2	468	148	0.83 (0.51-1.34)	0.35 (-4.93-5.62)	1.87 (-11.02-14.77)
	IIB	3059	1159	0.92 (0.74-1.15)	0.71 (-1.85-3.26)	1.86 (-4.50-8.23)
*Propensity score-matched **						
Included	IB3	504	140	0.70 (0.50-0.97)	3.60 (0.53-6.68)	8.63 (1.04-16.21)
	IIA2	158	58	0.75 (0.42-1.31)	3.14 (-3.70-9.98)	7.86 (-8.67-24.39)
	IIB	701	280	0.89 (0.69-1.15)	2.27 (-0.83-5.38)	4.66 (-2.98-12.30)
Excluded	IB3	375	106	0.57 (0.39-0.83)	4.49 (0.64-8.34)	11.30 (1.98-20.61)
	IIA2	147	57	0.66 (0.38-1.17)	4.62 (-2.18-11.43)	10.71 (-5.91-27.33)
	IIB	696	270	0.86 (0.65-1.12)	2.38 (-0.80-5.55)	5.09 (-2.73-12.91)
**Cancer-specific survival**						
All patients						
Included	IB3	1234	269	0.63 (0.47-0.84)	2.34 (0.34-4.34)	7.15 (2.13-12.17)
	IIA2	536	129	0.99 (0.59-1.68)	-0.16 (-5.25-4.92)	0.26 (-12.32-12.83)
	IIB	3288	905	0.96 (0.74-1.24)	0.40 (-2.08-2.87)	1.14 (-5.08-7.36)
Excluded	IB3	1001	235	0.52 (0.39-0.71)	3.86 (1.68-6.04)	11.10 (5.65-16.55)
	IIA2	468	115	0.93 (0.55-1.58)	0.04 (-5.10-5.19)	1.35 (-11.38-14.09)

	IIB	3059	826	0.97 (0.75-1.26)	0.34 (-2.14-2.82)	0.81 (-5.43-7.05)
Propensity score-matched						
	IB3	504	100	0.78 (0.52-1.15)	2.01 (-0.93-4.95)	5.24 (-2.11-12.60)
Included	IIA2	158	47	0.79 (0.42-1.47)	2.68 (-4.05-9.41)	7.93 (-8.46-24.31)
	IIB	701	204	0.89 (0.66-1.21)	1.83 (-1.18-4.84)	4.09 (-3.41-11.59)
	IB3	375	76	0.67 (0.43-1.05)	2.91 (-0.76-6.58)	7.08 (-1.93-16.10)
Excluded	IIA2	147	47	0.68 (0.37-1.28)	4.78 (-1.90-11.46)	12.03 (-4.53-28.58)
	IIB	696	199	0.90 (0.65-1.22)	1.77 (-1.31-4.86)	3.96 (-3.73-11.64)

* Patients in the radiotherapy group who underwent total hysterectomy were excluded prior to re-estimating the propensity scores and re-executing propensity score matching.
