# Supplementary material for: Comparison of Survival After Radical Hysterectomy or Radical Radiotherapy Among Patients with Different Histological Types of Locally Advanced Cervical Cancer: Analysis of SEER Data Using Propensity Score Matching
Source: Cancers (Basel). 2026 Oct 18;18(20):3371. doi: 10.3390/cancers18203371 (PMC13702493; doi:10.3390/cancers18203371)
Supplement: Supplementary file 1 [file cancers-18-03371-s001.zip › cancers-4586087-supplementary/Supplementary Tables/Supplementary Table S3.pdf]

Supplementary Table S3. Multivariate regression to identify independent predictors of overall and cancer-specific survival among individuals with squamous cell carcinoma, before and after propensity score matching.

Parameter	Overall survival				Cancer-specific survival			
	All patients		Propensity score-matched		All patients		Propensity score-matched	
	HR (95% CI)	p	HR (95% CI)	p	HR (95% CI)	p	HR (95% CI)	p
Treatment								
Radiotherapy	Reference		Reference		Reference		Reference	
Surgery	0.69 (0.58-0.83)	<0.001	0.80 (0.65-0.97)	0.025	0.73 (0.60-0.90)	0.003	0.84 (0.66-1.05)	0.131
Age at diagnosis, yr								
< 60	Reference		Reference		Reference		Reference	
≥ 60	1.46 (1.32-1.63)	<0.001	1.67 (1.34-2.08)	<0.001	1.01 (0.88-1.16)	0.840	1.25 (0.96-1.64)	0.097
Year of diagnosis								
2000-2014	Reference		Reference		Reference		Reference	
2015-2022	0.71 (0.62-0.81)	<0.001	0.79 (0.61-1.03)	0.083	0.63 (0.54-0.73)	<0.001	0.77 (0.58-1.03)	0.079
Ethnicity								
White	Reference		Reference		Reference		Reference	
Black	1.16 (1.03-1.31)	0.016	1.30 (1.03-1.66)	0.030	1.06 (0.91-1.22)	0.463	1.15 (0.86-1.53)	0.345
Others	0.82 (0.70-0.96)	0.013	0.82 (0.60-1.12)	0.216	0.88 (0.73-1.05)	0.159	0.76 (0.52-1.10)	0.143
Tumor size, cm*								
< 4	Reference		Reference		Reference		Reference	
≥ 4	1.31 (1.12-1.53)	<0.001	1.72 (1.22-2.42)	0.002	1.41 (1.17-1.70)	<0.001	1.65 (1.23-2.21)	<0.001
Unknown	1.37 (1.16-1.61)	<0.001	1.13 (0.71-1.81)	0.602	1.38 (1.13-1.69)	0.001	1.39 (0.96-2.01)	0.084
FIGO stage								
IB3	Reference		Reference		Reference		Reference	
IIA2	1.28 (1.10-1.50)	0.002	1.51 (1.14-2.01)	0.004	1.39 (1.15-1.67)	<0.001	1.58 (1.13-2.20)	0.007
IIB	1.33 (1.13-1.57)	<0.001	1.33 (0.92-1.91)	0.129	1.34 (1.10-1.64)	0.003	1.08 (0.68-1.71)	0.745
Tumor grade								
1	Reference		Reference		Reference		Reference	
2	1.14 (0.85-1.53)	0.380	0.47 (0.21-1.07)	0.073	1.44 (0.98-2.13)	0.064	0.42 (0.17-1.03)	0.059
3-4	1.38 (1.03-1.85)	0.032	0.54 (0.24-1.22)	0.139	1.76 (1.19-2.59)	0.004	0.46 (0.19-1.13)	0.089
Unknown	1.21 (0.90-1.63)	0.207	0.47 (0.19-1.16)	0.101	1.45 (0.98-2.15)	0.064	0.29 (0.10-0.80)	0.018
Chemotherapy								
Yes	Reference		Reference		Reference		Reference	
No/Unknown	1.25 (1.09-1.43)	0.001	1.27 (1.05-1.54)	0.013	1.15 (0.98-1.36)	0.090	1.35 (1.08-1.68)	0.008

CI, confidence interval; HR, hazard ratio.
* This analysis was restricted to patients with stage IIB disease.
