# Supplementary material for: Comparison of Survival After Radical Hysterectomy or Radical Radiotherapy Among Patients with Different Histological Types of Locally Advanced Cervical Cancer: Analysis of SEER Data Using Propensity Score Matching
Source: Cancers (Basel). 2026 Oct 18;18(20):3371. doi: 10.3390/cancers18203371 (PMC13702493; doi:10.3390/cancers18203371)
Supplement: Supplementary file 1 [file cancers-18-03371-s001.zip › cancers-4586087-supplementary/Supplementary Tables/Supplementary Table S4.pdf]

Supplementary Table S4. Sensitivity analysis to assess interaction of histological type of cervical cancer (adenocarcinoma or adenosquamous carcinoma) with treatment type on survival.

Outcome, subgroup and histological type	Number of		Hazard ratio *	Difference in restricted mean survival time, months	
	Patients	Events		At 60 months	At 120 months
**Cancer-specific survival**					
All patients					
Adenocarcinoma	1,011	295	0.41 (0.29-0.60)	6.38 (4.46 to 8.30)	16.59 (10.95 to 22.24)
Adenosquamous carcinoma	334	121	0.46 (0.28-0.75)	7.09 (2.99 to 11.19)	17.83 (7.45 to 28.20)
p for interaction			p = 0.845		
Propensity score-matched					
Adenocarcinoma	417	116	0.50 (0.32-0.78)	6.49 (3.61 to 9.36)	14.42 (6.21 to 22.63)
Adenosquamous carcinoma	159	48	0.58 (0.32-1.06)	3.93 (-1.51 to 9.36)	10.09 (-3.24 to 23.41)
p for interaction			p = 0.717		
**Overall survival**					
All patients					
Adenocarcinoma	1,011	356	0.45 (0.33-0.62)	6.76 (4.79 to 8.73)	16.73 (10.92 to 22.53)
Adenosquamous carcinoma	334	150	0.44 (0.28-0.68)	8.37 (4.09 to 12.65)	21.97 (11.44 to 32.51)
p for interaction			p = 0.789		
Propensity score-matched					
Adenocarcinoma	417	137	0.57 (0.38-0.84)	6.90 (3.97 to 9.83)	14.22 (5.87 to 22.56)
Adenosquamous carcinoma	159	59	0.55 (0.32-0.94)	5.62 (0.12 to 11.12)	15.34 (1.87 to 28.80)
p for interaction			p = 0.875		

Values are n or point estimates (95% confidence interval), unless otherwise noted.

* For the surgery arm relative to the radiotherapy arm.
