# Supplementary material for: Comparison of Survival After Radical Hysterectomy or Radical Radiotherapy Among Patients with Different Histological Types of Locally Advanced Cervical Cancer: Analysis of SEER Data Using Propensity Score Matching
Source: Cancers (Basel). 2026 Oct 18;18(20):3371. doi: 10.3390/cancers18203371 (PMC13702493; doi:10.3390/cancers18203371)
Supplement: Supplementary file 1 [file cancers-18-03371-s001.zip › cancers-4586087-supplementary/Supplementary Tables/Supplementary Table S5.pdf]

Supplementary Table S5. Sensitivity analysis of treatment effect on survival in adenocarcinoma: including vs. excluding patients with total hysterectomy in the radiotherapy group.

Outcome, subgroup, and hysterectomy in the radiotherapy group	FIGO stage	Number of		Hazard ratio	Difference in restricted mean survival time, months	
		Patients	Events		At 60 months	At 120 months
**Overall survival**						
All patients						
Included	IB3	456	140	0.42 (0.28-0.62)	4.89 (2.18-7.60)	14.53 (7.32-21.73)
	IIA2	148	45	0.53 (0.24-1.19)	5.08 (-0.96-11.12)	13.60 (-1.93-29.13)
	IIB	741	321	0.66 (0.44-0.98)	6.94 (3.62-10.26)	12.49 (2.72-22.26)
Excluded	IB3	362	105	0.39 (0.26-0.60)	5.80 (2.66-8.95)	16.78 (8.51-25.05)
	IIA2	113	34	0.48 (0.21-1.11)	5.49 (-1.02-12.01)	16.08 (-0.70-32.85)
	IIB	627	280	0.62 (0.42-0.92)	7.92 (4.51-11.34)	14.35 (4.40-24.29)
*Propensity score-matched **						
Included	IB3	237	68	0.46 (0.28-0.77)	4.46 (0.48-8.44)	12.97 (2.67-23.27)
	IIA2	75	26	0.38 (0.15-0.96)	8.69 (1.59-15.80)	23.84 (4.75-42.93)
	IIB	264	102	0.87 (0.56-1.36)	6.43 (2.28-10.59)	7.82 (-4.06-19.69)
Excluded	IB3	190	58	0.46 (0.27-0.79)	5.25 (0.55-9.95)	15.72 (3.62-27.82)
	IIA2	63	20	0.36 (0.13-0.99)	7.39 (0.49-14.30)	23.14 (3.69-42.60)
	IIB	235	99	0.71 (0.44-1.15)	7.53 (3.28-11.78)	12.28 (-0.27-24.84)
**Cancer-specific survival**						
All patients						
Included	IB3	456	108	0.47 (0.30-0.73)	4.39 (1.75-7.04)	12.88 (5.86-19.90)
	IIA2	148	36	0.27 (0.08-0.88)	6.24 (1.08-11.40)	18.34 (4.46-32.21)
	IIB	741	272	0.64 (0.41-0.99)	5.88 (2.57-9.19)	10.50 (0.75-20.26)
Excluded	IB3	362	82	0.44 (0.28-0.70)	5.25 (2.17-8.34)	15.08 (7.00-23.15)
	IIA2	113	27	0.24 (0.07-0.81)	6.92 (1.18-12.65)	20.83 (5.58-36.09)

	IIB	627	236	0.6 (0.39-0.94)	6.71 (3.31-10.11)	12.01 (2.08-21.93)
Propensity score-matched						
	IB3	237	55	0.51 (0.29-0.90)	3.66 (-0.22-7.55)	10.73 (0.67-20.79)
Included	IIA2	75	21	0.22 (0.07-0.76)	8.28 (1.15-15.40)	24.51 (5.89-43.13)
	IIB	264	88	0.79 (0.48-1.29)	5.56 (1.44-9.67)	6.58 (-5.27-18.43)
	IB3	190	48	0.53 (0.29-0.95)	4.25 (-0.41-8.91)	12.45 (0.45-24.45)
Excluded	IIA2	63	16	0.16 (0.04-0.71)	7.39 (0.49-14.30)	25.82 (7.00-44.64)
	IIB	235	87	0.68 (0.40-1.13)	6.61 (2.41-10.81)	10.94 (-1.53-23.41)

* Patients in the radiotherapy group who underwent total hysterectomy were excluded prior to re-estimating the propensity scores and re-executing propensity score matching.
