# Supplementary material for: Comparison of Survival After Radical Hysterectomy or Radical Radiotherapy Among Patients with Different Histological Types of Locally Advanced Cervical Cancer: Analysis of SEER Data Using Propensity Score Matching
Source: Cancers (Basel). 2026 Oct 18;18(20):3371. doi: 10.3390/cancers18203371 (PMC13702493; doi:10.3390/cancers18203371)
Supplement: Supplementary file 1 [file cancers-18-03371-s001.zip › cancers-4586087-supplementary/Supplementary Tables/Supplementary Table S6.pdf]

Supplementary Table S6. Multivariate regression to identify independent predictors of overall and cancer-specific survival among individuals with adenocarcinoma, before and after propensity score matching.

Parameter	Overall survival				Cancer-specific survival			
	All patients		Propensity score-matched		All patients		Propensity score-matched	
	HR (95% CI)	p	HR (95% CI)	p	HR (95% CI)	p	HR (95% CI)	p
Treatment								
Radiotherapy	Reference		Reference		Reference		Reference	
Surgery	0.60 (0.45-0.81)	<0.001	0.64 (0.46-0.89)	0.008	0.62 (0.45-0.85)	0.004	0.61 (0.42-0.89)	0.009
Age at diagnosis, yr								
< 60	Reference		Reference		Reference		Reference	
≥ 60	1.77 (1.45-2.16)	<0.001	1.70 (1.19-2.41)	0.003	1.32 (1.04-1.67)	0.022	1.34 (0.89-2.03)	0.161
Year of diagnosis								
2000-2014	Reference		Reference		Reference		Reference	
2015-2022	0.82 (0.65-1.04)	0.095	0.79 (0.53-1.17)	0.236	0.75 (0.58-0.97)	0.030	0.71 (0.46-1.08)	0.108
Ethnicity								
White	Reference		Reference		Reference		Reference	
Black	1.96 (1.51-2.52)	<0.001	1.99 (1.31-3.03)	0.001	1.88 (1.41-2.51)	<0.001	1.85 (1.16-2.96)	0.010
Others	0.99 (0.76-1.29)	0.920	0.97 (0.62-1.50)	0.879	1.00 (0.75-1.33)	0.992	0.91 (0.56-1.48)	0.709
Tumor size, cm *								
< 4	Reference		Reference		Reference		Reference	
≥ 4	1.36 (1.01-1.83)	0.045	1.26 (0.78-2.02)	0.346	1.39 (1.00-1.92)	0.049	1.43 (0.85-2.40)	0.182
Unknown	1.62 (1.18-2.24)	0.003	2.02 (0.96-4.22)	0.062	1.59 (1.12-2.27)	0.009	2.25 (1.02-4.95)	0.044
FIGO stage								
IB3	Reference		Reference		Reference		Reference	
IIA2	1.23 (0.92-1.65)	0.166	1.08 (0.69-1.68)	0.745	1.26 (0.92-1.74)	0.149	1.21 (0.74-1.97)	0.447
IIB	1.61 (1.19-2.19)	0.002	1.97 (1.03-3.75)	0.039	1.62 (1.16-2.26)	0.005	2.36 (1.19-4.69)	0.014
Tumor grade								
1	Reference		Reference		Reference		Reference	
2	1.41 (1.02-1.95)	0.036	1.42 (0.87-2.33)	0.162	1.44 (1.00-2.05)	0.048	1.18 (0.70-2.00)	0.528
3-4	1.72 (1.25-2.36)	<0.001	1.82 (1.11-2.96)	0.017	1.73 (1.22-2.47)	0.002	1.70 (1.01-2.84)	0.044
Unknown	1.58 (1.13-2.19)	0.007	1.72 (0.89-3.34)	0.108	1.54 (1.07-2.22)	0.022	1.56 (0.76-3.20)	0.221
Chemotherapy								
Yes	Reference		Reference		Reference		Reference	
No/Unknown	0.93 (0.72-1.20)	0.585	0.75 (0.52-1.08)	0.118	0.76 (0.56-1.03)	0.073	0.64 (0.43-0.98)	0.038

CI, confidence interval; HR, hazard ratio.
* This analysis was restricted to patients with stage IIB disease.
